# Supplementary material for: STIM1–Orai1 Interaction Exacerbates LPS-Induced Inflammation and Endoplasmic Reticulum Stress in Bovine Hepatocytes through Store-Operated Calcium Entry
Source: Genes (Basel). 2022 May 13;13(5):874. doi: 10.3390/genes13050874 (PMC9140735; doi:10.3390/genes13050874)
Supplement: Supplementary file 1 [file genes-13-00874-s001.zip › genes-1701846-supplementary.pdf]

Supplemental data

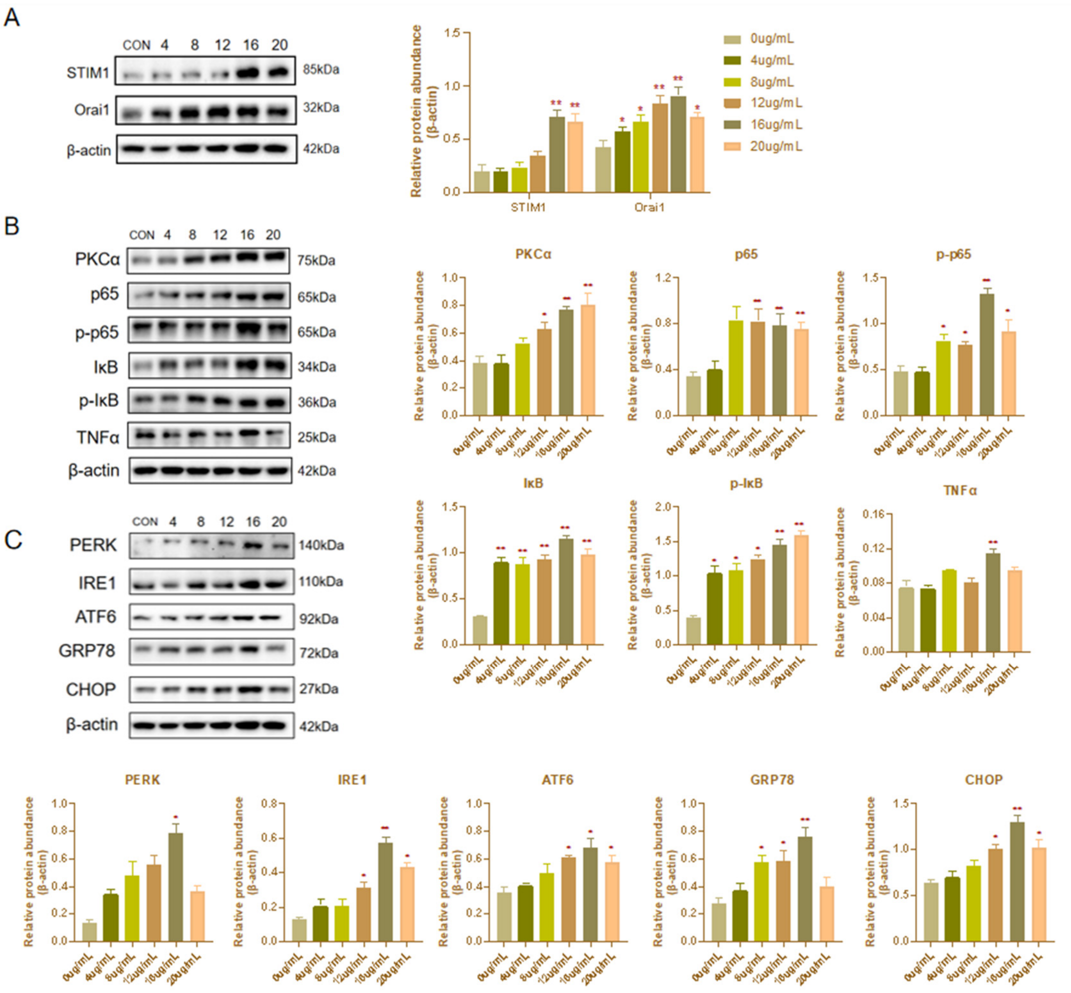

Figure S1. Protein abundance of bovine hepatocytes (BHEC) exposed to different concentrations of LPS. When the adherent density of BHEC reached about 70%, the BHEC was treated with LPS at 0, 4, 8, 12, 16, or 20 μg/mL for 12 h. Protein abundance was corrected with β-actin as the reference protein. (A) Protein abundance of STIM1 and Orai1 in BHEC. (B) Proteins (PKCα, p65, p-p65, IκB, p-IκB and TNFα) expression levels were detected. (C) Changes in endoplasmic reticulum stress (ERS)-related proteins (PERK, IRE1, ATF6, GRP78, and CHOP) under different doses of LPS. Error bars represent the means ± SEM (n = 3, n = 3). **P*<0.05, ***P* < 0.01 compared with controls.

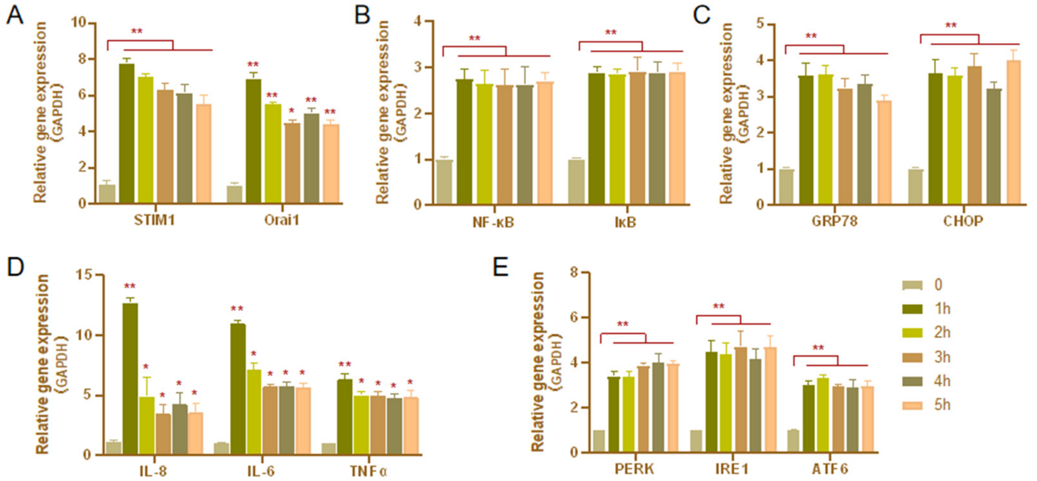

Figure S2. Effects of LPS on STIM1/Orai1, inflammation, and endoplasmic reticulum stress (ERS) in BHEC at different time points. (A-E) BHEC were exposed to 16 μg/mL LPS for 0, 1, 2, 3, 4, or 5 h. The expression levels of STIM1/Orai1 (A), the genes related to the NF-κB signaling pathway (NF-κB, IκB) (B), inflammatory cytokines (IL-6, IL-8, and TNFα) (D) and ERS (PERK, IRE1, ATF6, GRP78, and CHOP) (C, E) are shown. Gene expression was corrected with GAPDH. The results are presented as the means ± SEM. *P < 0.05; **P < 0.01.

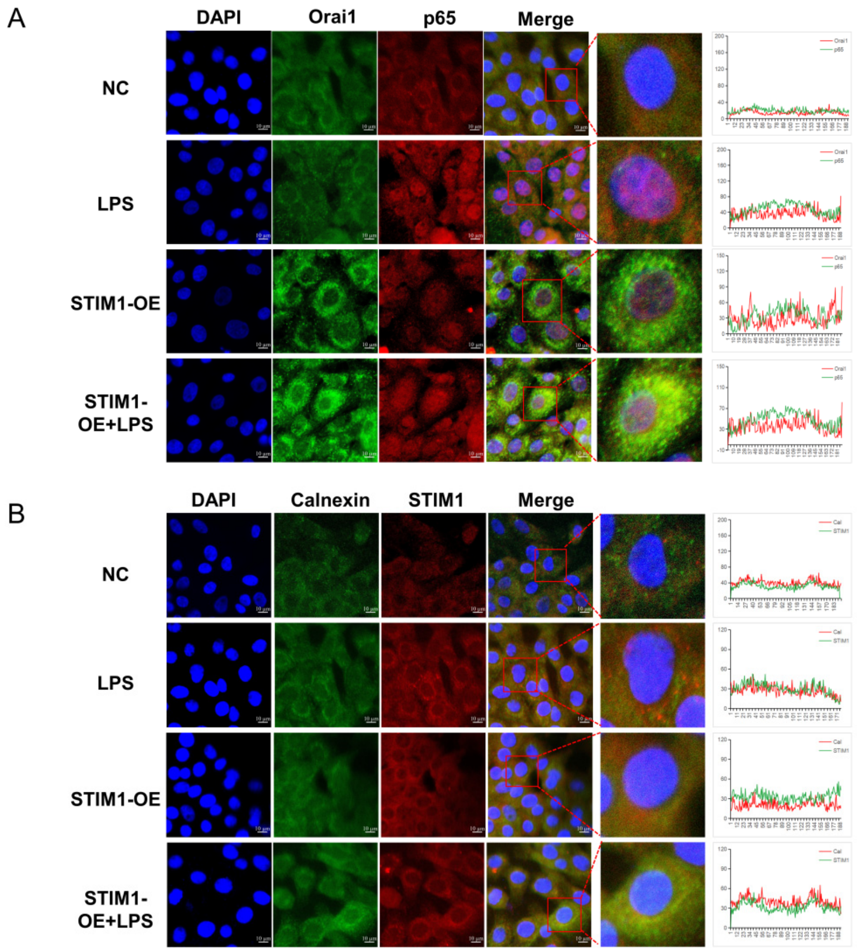

Figure S3. Protein expression and location of STIM1/Orai1, NF-κB p65, and calnexin protein in

BMEC. DAPI blue fluorescence was used to label the nuclear location. FITC green fluorescence was used to label Orai1 and calnexin protein, and Cy3 red fluorescence was used to label p65 and STIM1 protein. Image J was used to draw the line graph to reflect the fluorescence co-location trends.

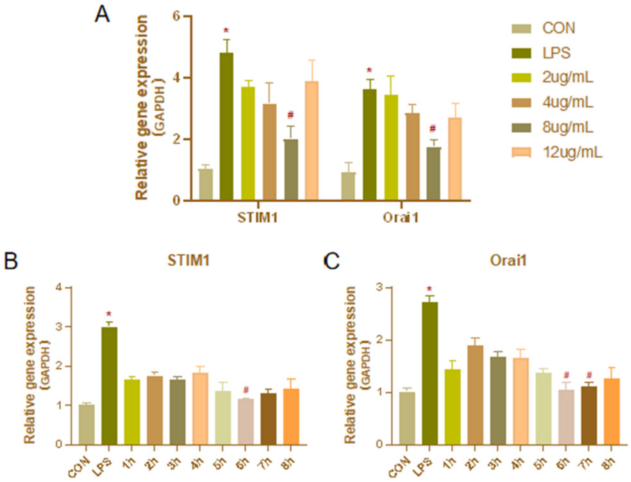

Figure S4. Effects of different doses and times of BTP2 pretreatment on LPS-induced inflammation and endoplasmic reticulum stress (ERS) in BHEC. (A) BHEC were pretreated with 0, 2, 4, 8, or 12 μg/mL BTP2 for 12 h and then exposed to 16 μg/mL LPS. (B, C) BHEC were pretreated with 8 μg/mL BTP2 for 0, 1, 2, 3, 4, 5, 6, 7, or 8 h. Gene expression was normalized to that of GAPDH. The results are presented as the means ± SEM. **P* < 0.05 and ***P* < 0.01 indicate a significant difference between the LPS groups and the control groups. $^{\#}P < 0.05$ and $^{\#\#}P<0.01$ indicates a significant difference between the BTP2 pretreatment groups and the LPS groups.

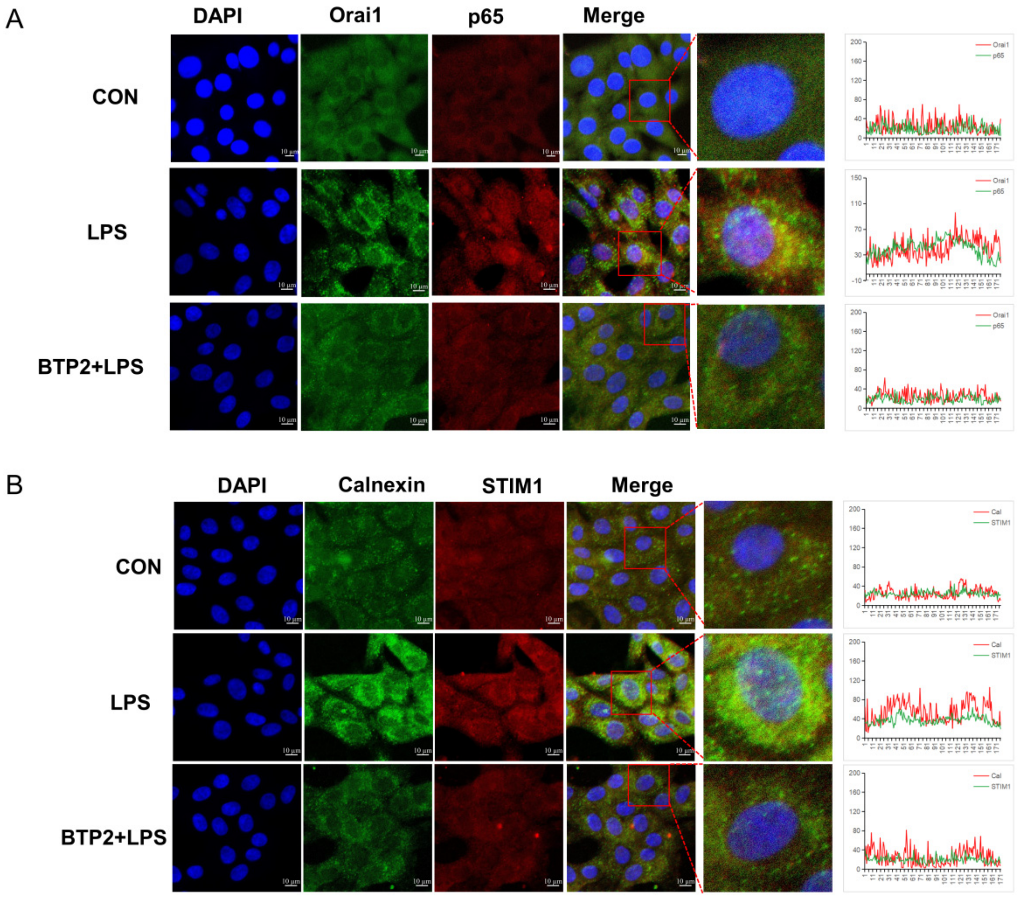

Figure S5. Protein expression and location of STIM1/Orai1, NF-κB p65 and calnexin protein in BMECs. DAPI blue fluorescence was used to label the nuclear location. FITC green fluorescence was used to label Orai1 and Calnexin protein, and Cy3 red fluorescence was used to label p65 and STIM1 protein. Image J was used to draw the line graph to reflect the fluorescence co-location trends.
